# Supplementary material for: DrugOn: a fully integrated pharmacophore modeling and structure optimization toolkit
Source: PeerJ. 2015 Jan 13;3:e725. doi: 10.7717/peerj.725 (PMC4304849; doi:10.7717/peerj.725)
Supplement: Supplemental Information 1 [file peerj-03-725-s001.zip › DrugOn_setup/Program_Manual/DrugOn_quick_guide.pdf]

A Quick Guide To
DrugOn

This is a brief description of DrugOn. DrugOn is a fully integrated pharmacophore modeling and structure optimization toolkit. For more information please visit :

www.bioacademy.gr/bioinformatics/drugon/

DrugOn overview

The DrugOn is fully interactive pipeline design to exploit the advantages of modern programming and overcome the command line barrier with 2 friendly environments for the user to perform pharmacophore modeling through an efficient combination of the pharmACOphore, Gromacs, Ligbuilder and PDB2PQR suites. You can download them (for the DrugOn setup) from the following links:http://www.ligbuilder.org/ http://sourceforge.net/projects/pdb2pqr/files/pdb2pqr/pdb2pqr-1.8/ http://www.tcd.uni-konstanz.de/research/pharmacophore.php

DrugOn

DrugOn’s main window is a frame layout at the left of the main window with one “next” and one “previous” button. The DrugOn’s pipeline also has four basic steps: Input preparations, receptor optimization, build ligands and pharmacophore.

Input preparation:

Input PDB file: input the path of the pdb to be analyzed.
Output PDB file: input the path and the name of the new (analyzed) pdb file.
Input preparation: starts the analysis of the input file.
Abort: interrupts the process.
Next: moves you to the next step (receptor optimization).

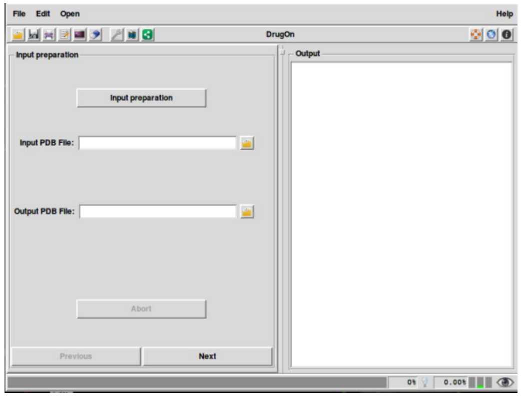

Receptor optimization:

Input PDB file: input the path of the pdb to be analyzed (the output of the previous step).
Output PDB file: input the path and the name of the new (analyzed) pdb file.
Receptor optimization: starts the analysis of the input file.
Abort: interrupts the process.
Next: moves you to the next step (Build ligands).
Previous: moves you to the previous step (Input preparation).

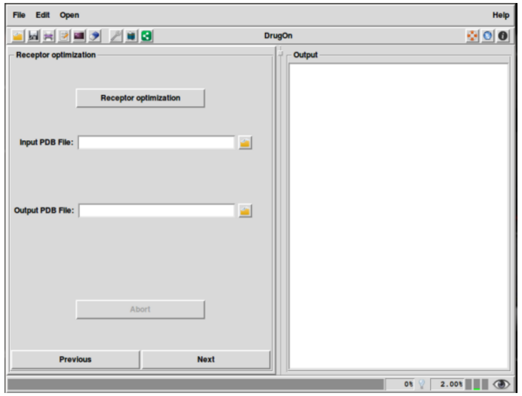

Build ligands:

Parameter File: input the path of the parameter file (you can also edit an already existed or create a new one) that will be used to pocket, grow, link or process respectively.
Pocket: starts the pocket process.
Grow: starts the grow process.
Link: starts the link process.
Process: starts the process.
Abort: interrupts the process.
Next: moves you to the next step

(Pharmacophore).
Previous: moves you to the previous step (Receptor optimization).

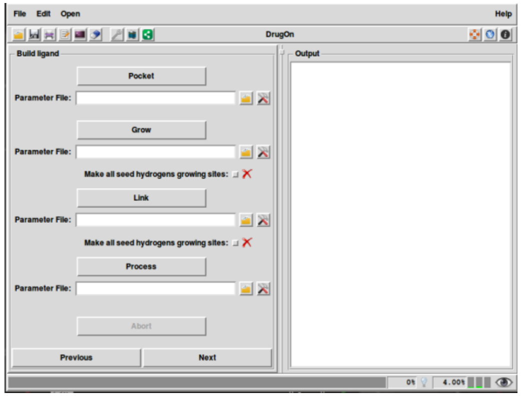

Pharmacophore:

Input mol2 Protein file: input the path of the mol2 protein to be analyzed.
Input mol2 Ligand file: input the path of the mol2 ligand to be analyzed (the output of the previous step).
Input files: inputs the protein and the ligand mol2 files into the DrugOn system.
Input Protein File name (.mol2): input the name of the mol2 protein to the configuration file (the name must be the same with the file that you input to the DrugOns system with the **Input mol2 Protein file)** .
Input Ligand File name (.mol2): input the name of the mol2 ligand to the configuration file (the name must be the same with the file that you input to the DrugOns system with the **Input mol2 Ligand file)**.
Output Pharmacophore File name: name the folder of the output files (for the configuration file)
Create file: adjusts the configuration file with the three previous parameters (Input Protein File name (.mol2), nput Ligand File name (.mol2), Output Pharmacophore File name)
Start the Construct: starts the pharmacophore construction.
Move: Moves the output (results) file that you named before (Output Pharmacophore File name) to a location of your choice (**directory**).
Abort: interrupts the process.
Previous: moves you to the previous step (Build ligands).

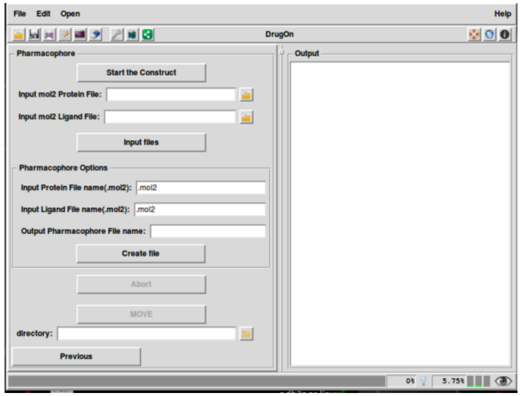

DrugOn

A quick guide
