# Supplementary figures and images for: DrugOn: a fully integrated pharmacophore modeling and structure optimization toolkit
Source: PeerJ. 2015 Jan 13;3:e725. doi: 10.7717/peerj.725 (PMC4304849; doi:10.7717/peerj.725)

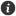

Supplement: Supplemental Information 1 [file peerj-03-725-s001.zip › DrugOn_setup/Icons/about.png]

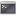

Supplement: Supplemental Information 1 [file peerj-03-725-s001.zip › DrugOn_setup/Icons/application-terminal.png]

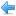

Supplement: Supplemental Information 1 [file peerj-03-725-s001.zip › DrugOn_setup/Icons/arrow-180.png]

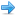

Supplement: Supplemental Information 1 [file peerj-03-725-s001.zip › DrugOn_setup/Icons/arrow.png]

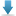

Supplement: Supplemental Information 1 [file peerj-03-725-s001.zip › DrugOn_setup/Icons/Arrow_Down.png]

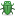

Supplement: Supplemental Information 1 [file peerj-03-725-s001.zip › DrugOn_setup/Icons/bug.png]

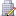

Supplement: Supplemental Information 1 [file peerj-03-725-s001.zip › DrugOn_setup/Icons/building--pencil.png]

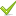

Supplement: Supplemental Information 1 [file peerj-03-725-s001.zip › DrugOn_setup/Icons/check.png]

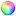

Supplement: Supplemental Information 1 [file peerj-03-725-s001.zip › DrugOn_setup/Icons/color.png]

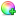

Supplement: Supplemental Information 1 [file peerj-03-725-s001.zip › DrugOn_setup/Icons/colorplus.png]

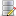

Supplement: Supplemental Information 1 [file peerj-03-725-s001.zip › DrugOn_setup/Icons/database--pencil.png]

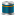

Supplement: Supplemental Information 1 [file peerj-03-725-s001.zip › DrugOn_setup/Icons/Database.png]

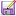

Supplement: Supplemental Information 1 [file peerj-03-725-s001.zip › DrugOn_setup/Icons/disk--pencil.png]

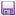

Supplement: Supplemental Information 1 [file peerj-03-725-s001.zip › DrugOn_setup/Icons/disk.png]

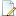

Supplement: Supplemental Information 1 [file peerj-03-725-s001.zip › DrugOn_setup/Icons/document--pencil.png]

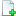

Supplement: Supplemental Information 1 [file peerj-03-725-s001.zip › DrugOn_setup/Icons/document--plus.png]

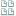

Supplement: Supplemental Information 1 [file peerj-03-725-s001.zip › DrugOn_setup/Icons/document-view.png]

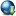

Supplement: Supplemental Information 1 [file peerj-03-725-s001.zip › DrugOn_setup/Icons/download.png]

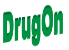

Supplement: Supplemental Information 1 [file peerj-03-725-s001.zip › DrugOn_setup/Icons/DrugOn-small.png]

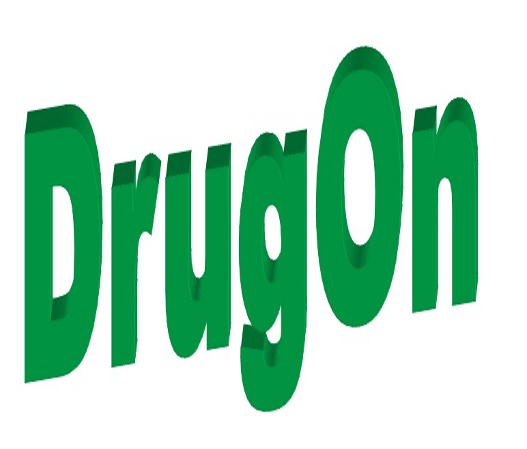

Supplement: Supplemental Information 1 [file peerj-03-725-s001.zip › DrugOn_setup/Icons/DrugOn.png]

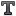

Supplement: Supplemental Information 1 [file peerj-03-725-s001.zip › DrugOn_setup/Icons/edit.png]

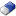

Supplement: Supplemental Information 1 [file peerj-03-725-s001.zip › DrugOn_setup/Icons/eraser.png]

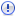

Supplement: Supplemental Information 1 [file peerj-03-725-s001.zip › DrugOn_setup/Icons/exclamation-white.png]

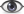

Supplement: Supplemental Information 1 [file peerj-03-725-s001.zip › DrugOn_setup/Icons/eye.png]

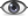

Supplement: Supplemental Information 1 [file peerj-03-725-s001.zip › DrugOn_setup/Icons/eye_b.png]

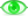

Supplement: Supplemental Information 1 [file peerj-03-725-s001.zip › DrugOn_setup/Icons/eye_b_on.png]

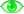

Supplement: Supplemental Information 1 [file peerj-03-725-s001.zip › DrugOn_setup/Icons/eye_on.png]

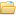

Supplement: Supplemental Information 1 [file peerj-03-725-s001.zip › DrugOn_setup/Icons/folder-horizontal-open.png]

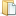

Supplement: Supplemental Information 1 [file peerj-03-725-s001.zip › DrugOn_setup/Icons/folder-open-document.png]

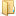

Supplement: Supplemental Information 1 [file peerj-03-725-s001.zip › DrugOn_setup/Icons/folder-open.png]

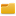

Supplement: Supplemental Information 1 [file peerj-03-725-s001.zip › DrugOn_setup/Icons/folder1.png]

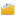

Supplement: Supplemental Information 1 [file peerj-03-725-s001.zip › DrugOn_setup/Icons/folder2.png]

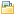

Supplement: Supplemental Information 1 [file peerj-03-725-s001.zip › DrugOn_setup/Icons/folder_images.gif]

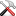

Supplement: Supplemental Information 1 [file peerj-03-725-s001.zip › DrugOn_setup/Icons/hammer-screwdriver.png]

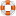

Supplement: Supplemental Information 1 [file peerj-03-725-s001.zip › DrugOn_setup/Icons/help.png]

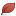

Supplement: Supplemental Information 1 [file peerj-03-725-s001.zip › DrugOn_setup/Icons/leaf-red.png]

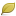

Supplement: Supplemental Information 1 [file peerj-03-725-s001.zip › DrugOn_setup/Icons/leaf-yellow.png]

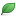

Supplement: Supplemental Information 1 [file peerj-03-725-s001.zip › DrugOn_setup/Icons/leaf.png]

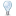

Supplement: Supplemental Information 1 [file peerj-03-725-s001.zip › DrugOn_setup/Icons/light-bulb-off.png]

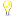

Supplement: Supplemental Information 1 [file peerj-03-725-s001.zip › DrugOn_setup/Icons/light-bulb.png]

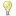

Supplement: Supplemental Information 1 [file peerj-03-725-s001.zip › DrugOn_setup/Icons/light-bulb_bak.png]

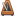

Supplement: Supplemental Information 1 [file peerj-03-725-s001.zip › DrugOn_setup/Icons/metronome.png]

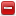

Supplement: Supplemental Information 1 [file peerj-03-725-s001.zip › DrugOn_setup/Icons/minus-button.png]

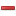

Supplement: Supplemental Information 1 [file peerj-03-725-s001.zip › DrugOn_setup/Icons/minus.png]

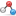

Supplement: Supplemental Information 1 [file peerj-03-725-s001.zip › DrugOn_setup/Icons/molecule.png]

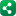

Supplement: Supplemental Information 1 [file peerj-03-725-s001.zip › DrugOn_setup/Icons/molecule1.png]

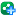

Supplement: Supplemental Information 1 [file peerj-03-725-s001.zip › DrugOn_setup/Icons/molecule1_plus.png]

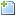

Supplement: Supplemental Information 1 [file peerj-03-725-s001.zip › DrugOn_setup/Icons/page_new.gif]

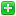

Supplement: Supplemental Information 1 [file peerj-03-725-s001.zip › DrugOn_setup/Icons/plus-button.png]

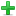

Supplement: Supplemental Information 1 [file peerj-03-725-s001.zip › DrugOn_setup/Icons/plus.png]

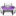

Supplement: Supplemental Information 1 [file peerj-03-725-s001.zip › DrugOn_setup/Icons/printer.png]

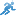

Supplement: Supplemental Information 1 [file peerj-03-725-s001.zip › DrugOn_setup/Icons/run_icon.png]

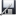

Supplement: Supplemental Information 1 [file peerj-03-725-s001.zip › DrugOn_setup/Icons/save.png]

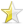

Supplement: Supplemental Information 1 [file peerj-03-725-s001.zip › DrugOn_setup/Icons/star-half.png]

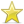

Supplement: Supplemental Information 1 [file peerj-03-725-s001.zip › DrugOn_setup/Icons/star.png]

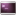

Supplement: Supplemental Information 1 [file peerj-03-725-s001.zip › DrugOn_setup/Icons/terminal.png]

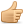

Supplement: Supplemental Information 1 [file peerj-03-725-s001.zip › DrugOn_setup/Icons/thumb-up.png]

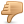

Supplement: Supplemental Information 1 [file peerj-03-725-s001.zip › DrugOn_setup/Icons/thumb.png]

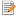

Supplement: Supplemental Information 1 [file peerj-03-725-s001.zip › DrugOn_setup/Icons/txt.png]

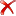

Supplement: Supplemental Information 1 [file peerj-03-725-s001.zip › DrugOn_setup/Icons/uncheck.png]

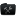

Supplement: Supplemental Information 1 [file peerj-03-725-s001.zip › DrugOn_setup/Icons/utilities.png]

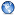

Supplement: Supplemental Information 1 [file peerj-03-725-s001.zip › DrugOn_setup/Icons/Website.png]

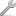

Supplement: Supplemental Information 1 [file peerj-03-725-s001.zip › DrugOn_setup/Icons/wrench.png]

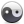

Supplement: Supplemental Information 1 [file peerj-03-725-s001.zip › DrugOn_setup/Icons/yin-yang.png]

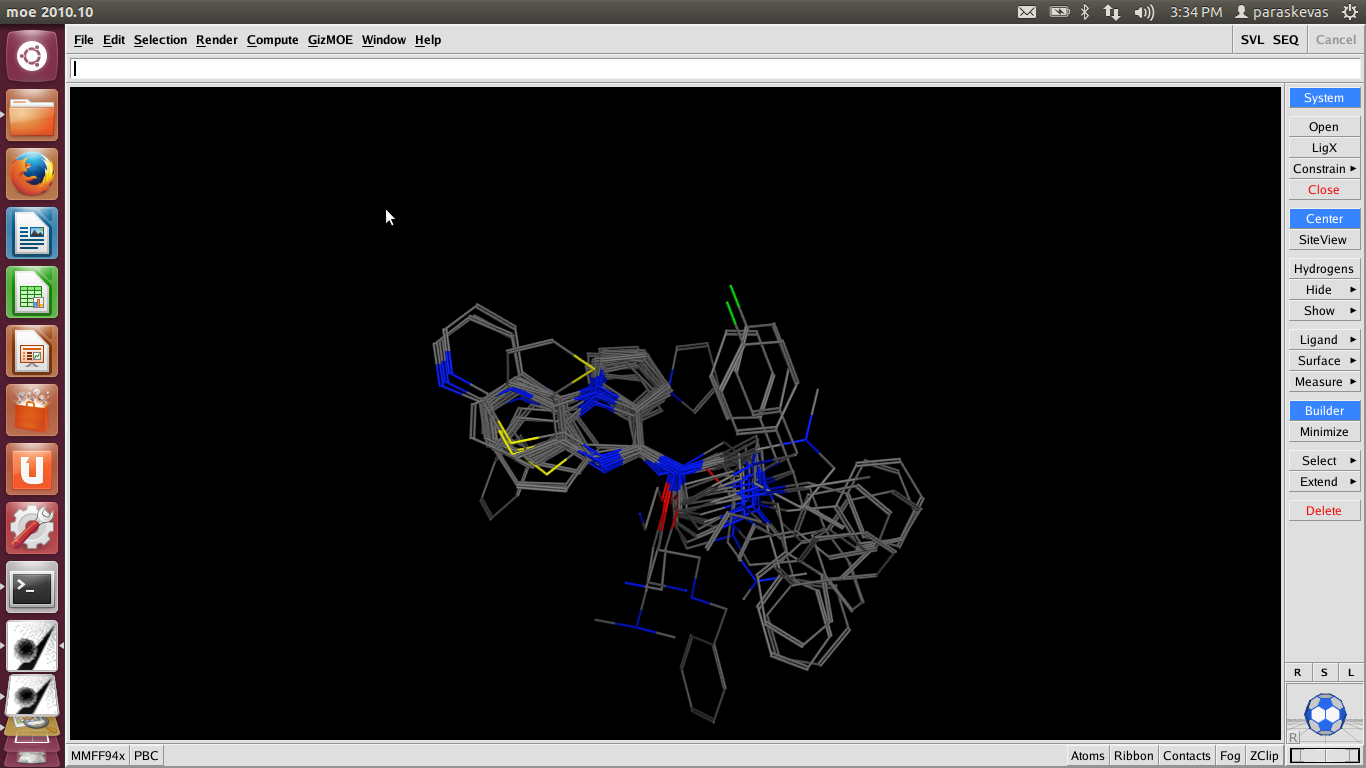

Supplement: Supplemental Information 3 — This is the raw dataset of the validation requested by the reviewers. [file peerj-03-725-s003.zip › Results for the validation/drugOn2.png]

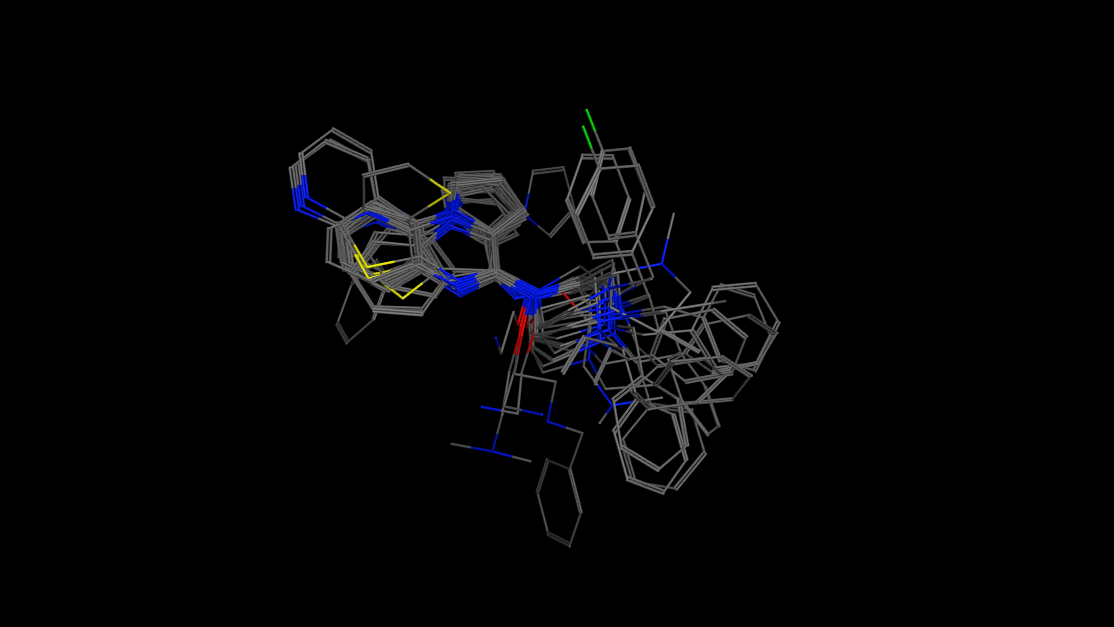

Supplement: Supplemental Information 3 — This is the raw dataset of the validation requested by the reviewers. [file peerj-03-725-s003.zip › Results for the validation/drugOn2b.png]

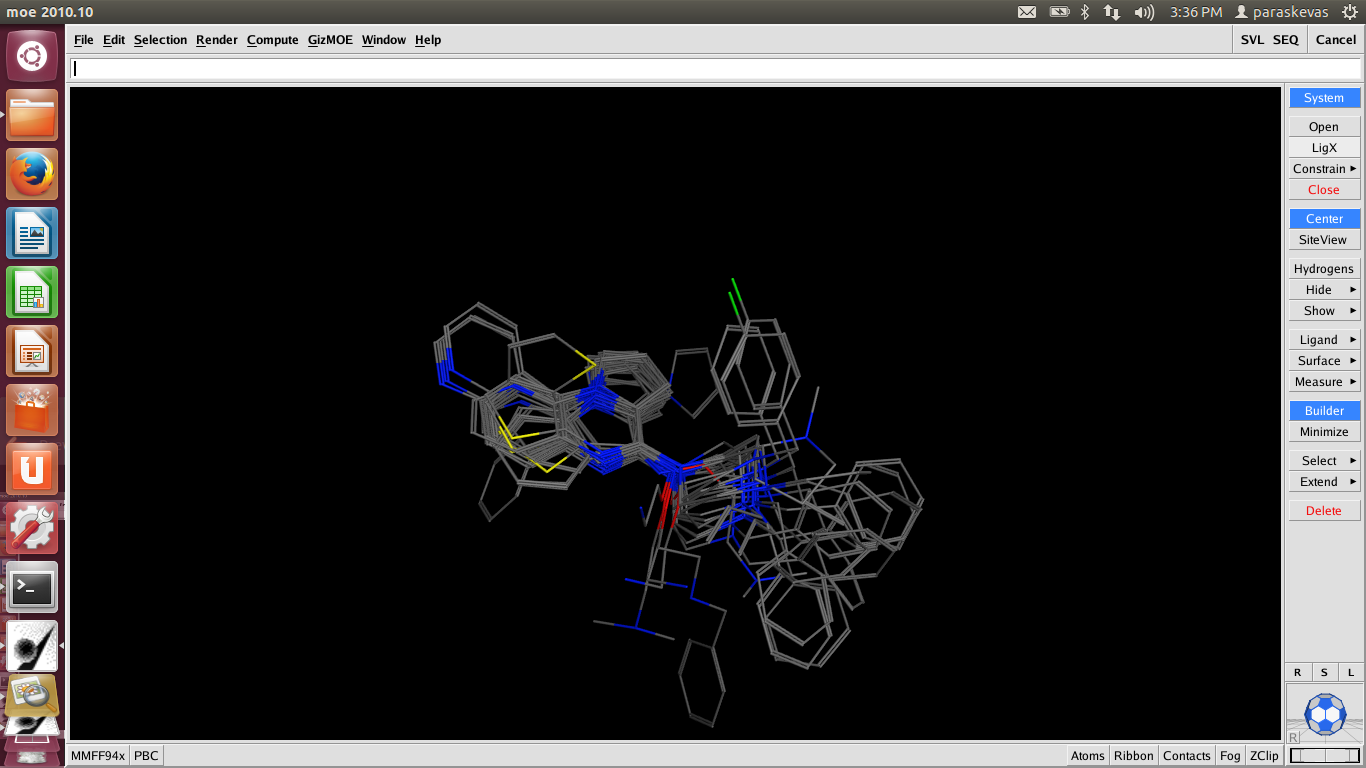

Supplement: Supplemental Information 3 — This is the raw dataset of the validation requested by the reviewers. [file peerj-03-725-s003.zip › Results for the validation/drugOn3.png]

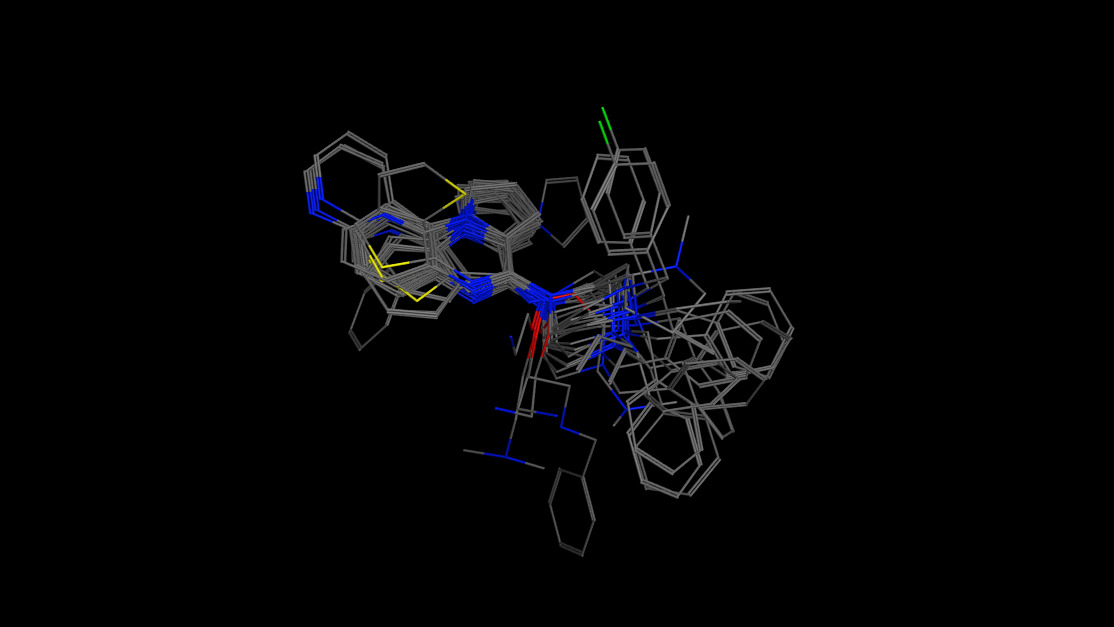

Supplement: Supplemental Information 3 — This is the raw dataset of the validation requested by the reviewers. [file peerj-03-725-s003.zip › Results for the validation/drugOn3b.png]

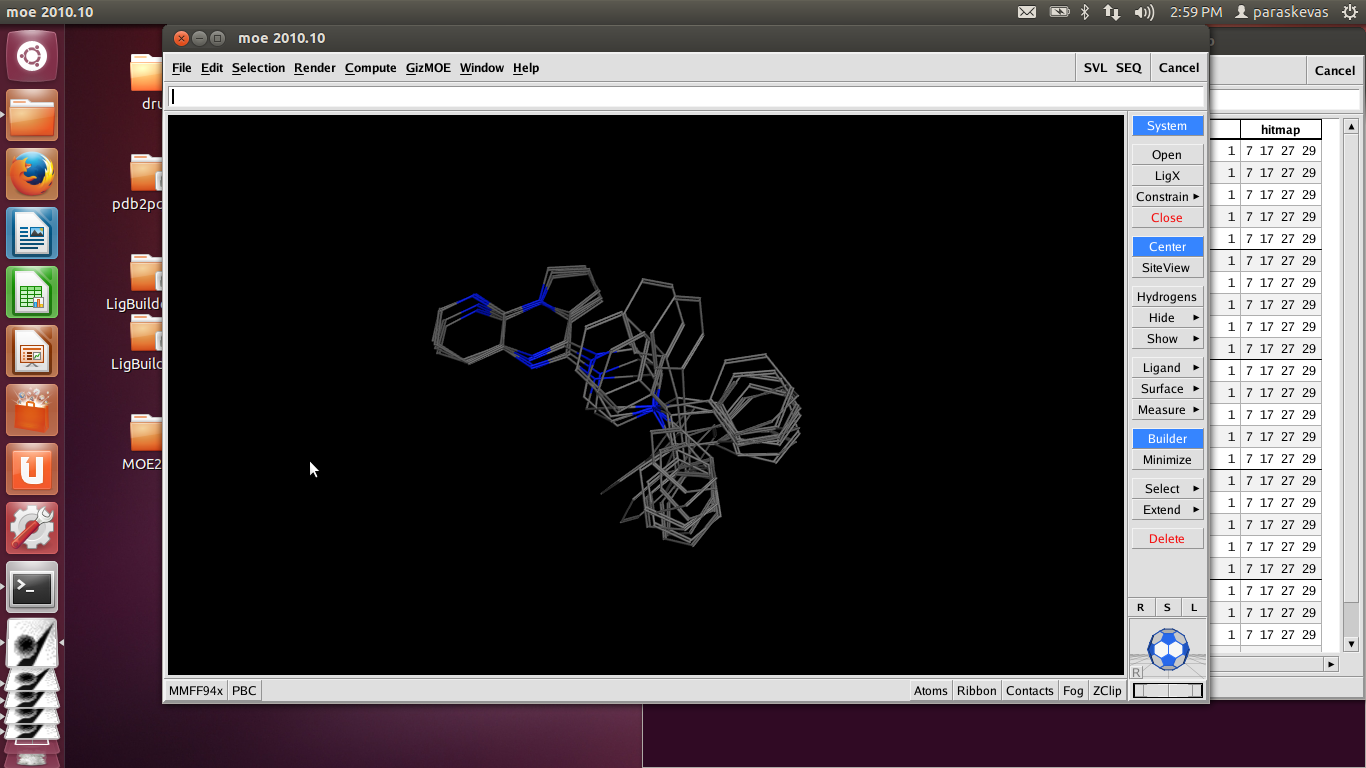

Supplement: Supplemental Information 3 — This is the raw dataset of the validation requested by the reviewers. [file peerj-03-725-s003.zip › Results for the validation/moe2.png]

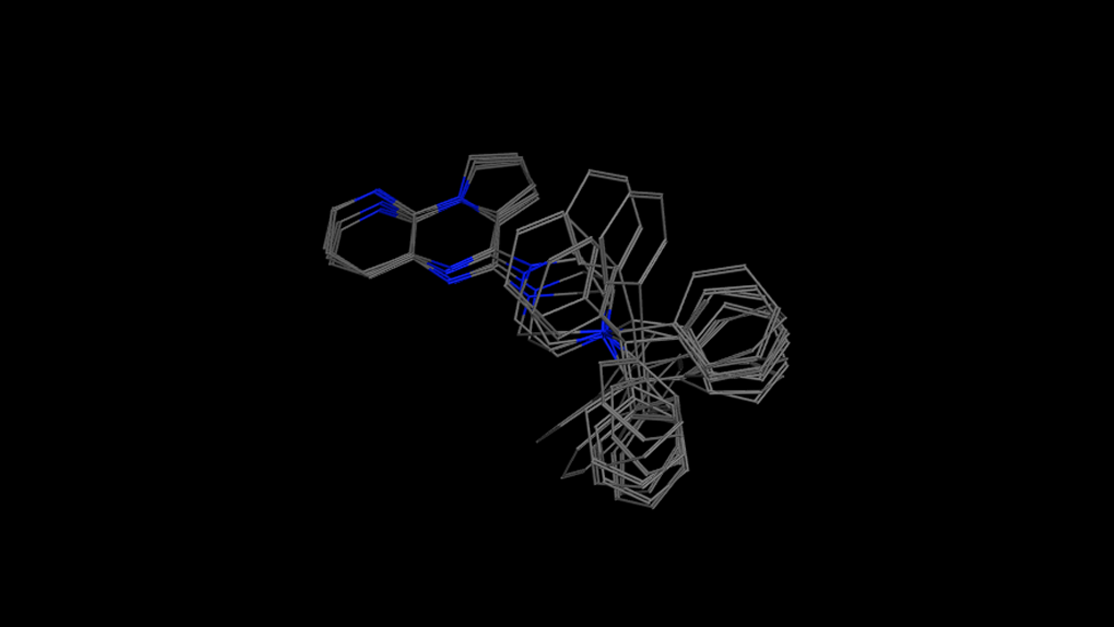

Supplement: Supplemental Information 3 — This is the raw dataset of the validation requested by the reviewers. [file peerj-03-725-s003.zip › Results for the validation/moe2b.png]

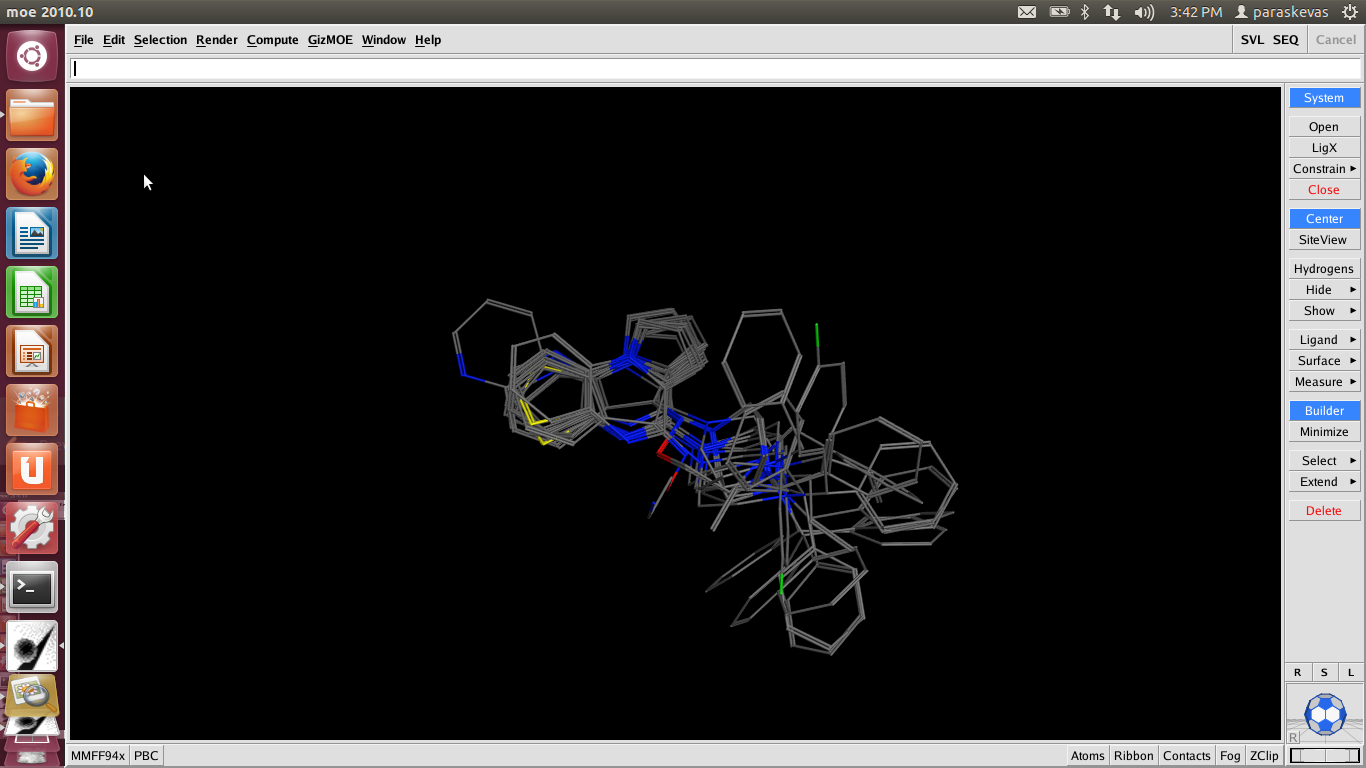

Supplement: Supplemental Information 3 — This is the raw dataset of the validation requested by the reviewers. [file peerj-03-725-s003.zip › Results for the validation/phase2.png]

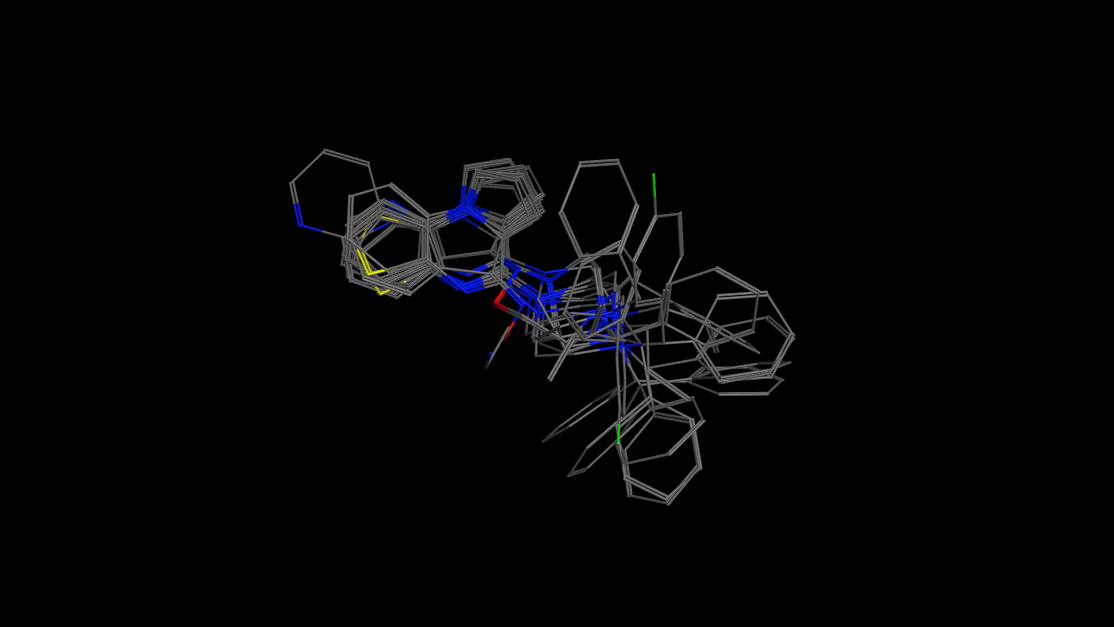

Supplement: Supplemental Information 3 — This is the raw dataset of the validation requested by the reviewers. [file peerj-03-725-s003.zip › Results for the validation/phase2b.png]

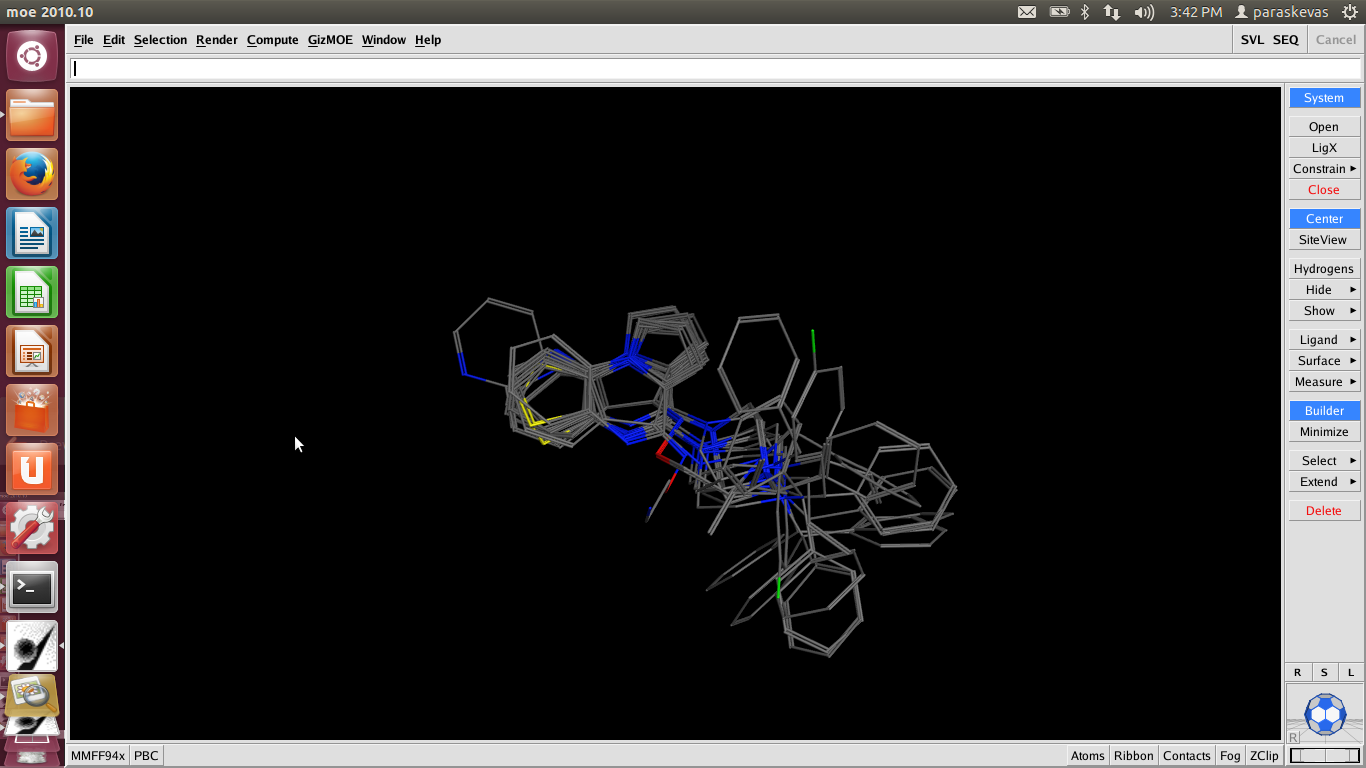

Supplement: Supplemental Information 3 — This is the raw dataset of the validation requested by the reviewers. [file peerj-03-725-s003.zip › Results for the validation/phase3.png]

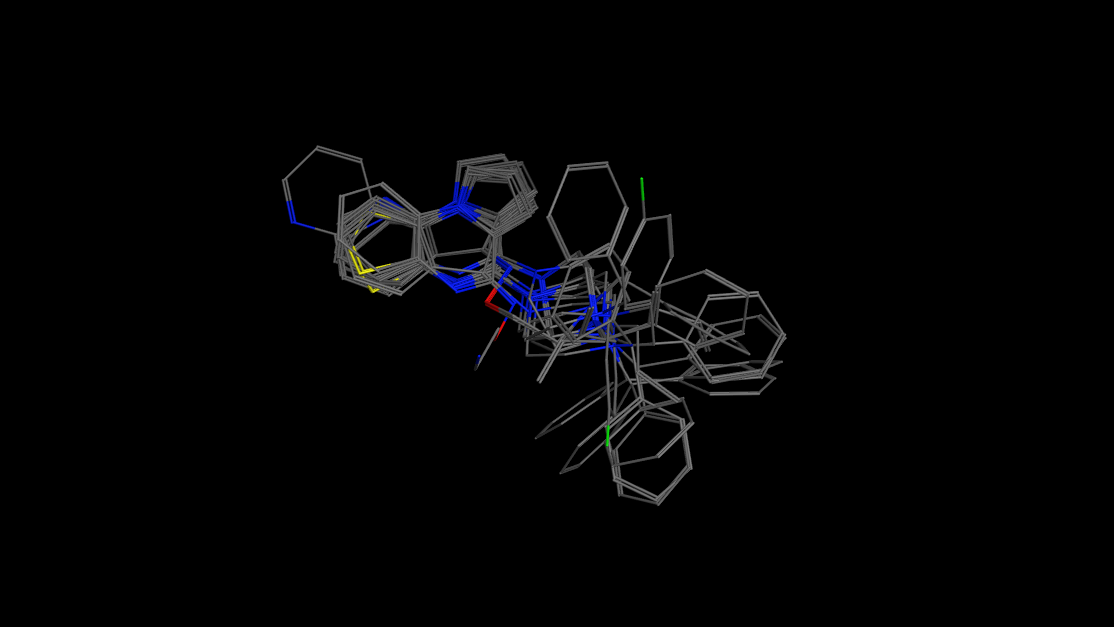

Supplement: Supplemental Information 3 — This is the raw dataset of the validation requested by the reviewers. [file peerj-03-725-s003.zip › Results for the validation/phase3b.png]
