# Supplementary material for: DrugOn: a fully integrated pharmacophore modeling and structure optimization toolkit
Source: PeerJ. 2015 Jan 13;3:e725. doi: 10.7717/peerj.725 (PMC4304849; doi:10.7717/peerj.725)
Supplement: Supplemental Information 1 [file peerj-03-725-s001.zip › DrugOn_setup/Supplementary_Files/dms/dms.html]

dms


#### NAME

### dms - calculate a molecular surface

#### SYNOPSIS

### **dms** *pdbfile* [ -a ] [ -d *density*] [ -g *logfile* ] [ -i *sitefile* ] [-n] [-w *radius* ] [-v] -o *outfile*

#### DESCRIPTION

**Dms** calculates the molecular surface of a molecule.
The molecular surface
resembles the van der Waals surface of a molecule, except that crevices
between atoms are smoothed over and interstices too small to accommodate
the probe are eliminated. The surface includes cavities in the interior of
the molecule, even if they are not accessible to a solvent molecule coming
from the outside.

The molecular surface calculated is that defined by Richards (see
the reference below).
In particular, the calculated molecular surface is that traced out by
the surface of the probe sphere rather that the
probe sphere's center. According to Richards' definition, the molecular
surface consists of two parts: contact surface and reentrant surface. The
contact surface is made up of "those parts of the molecular van der Waals
surface that can actually be in contact with the surface of the probe."
The reentrant surface is defined by "the interior-facing part of the probe
when it is simultaneously in contact with more than one atom." **Dms**
reports the amounts of contact and reentrant surface area, and the combined
total surface area on the standard error output (see 
**-g**).

*Pdbfile* is an input file of coordinates.
The input file must be in the Protein Data Bank format.
The first part of each atom
name is used to determine the element type. By default, implicit hydrogens
are included for carbon, nitrogen and oxygen atoms; thus, aromatic carbons
and nitrogens will have van der Waals radii that are somewhat too big.
Note that only amino acid and nucleic acid residues will be included unless
**-a** is also specified.

#### COMMAND-LINE ARGUMENTS

> **-a**
>   
> Include all atoms, not just those in amino acid and nucleic acid residues.

> **-d** *density*
>   
>  *Density* is a factor affecting the density of points
> on the surface; the default of **1.0**
> produces about 5 points per square angstrom. Only values between
> **0.1** and **10.0** are permitted.
> A density of **0.5** is recommended for large molecules.

> **-g** *logfile*
>   
>  Write all the informative messages to *logfile* instead of the
> standard error output. Genuine errors still go to the standard error
> output. New log information is appended to *logfile* rather than
> overwriting it, and thus messages from several runs may be accumulated.

> **-i** *sitefile*
>   
>  Calculate the molecular surface only for those residues and atoms
> specified in *sitefile*,
> keeping the rest of the molecule for collision checks.
> *Sitefile* consists of lines such as the following:
> > |  |  |  |
> > | --- | --- | --- |
> > | ASP | 205 | CA |
> > | TYR | 13 | \* |
> > | GLY | 116 | FRM |
> > | HIS | 178 | TO |
>
> The asterisk, **\***, means all atoms of the residue, and the
> "FRM" and "TO" lines mean all residues from 116 to 178 inclusive.
> The sequence number may
> contain letters, and if the PDB input file contains chain identifiers,
> then those should be appended on the right of the sequence number.
> Residue insertion codes (if any) should be placed between the sequence
> number and any chain identifier. Residues contained in HETATM records
> should have an asterisk appended to the end of the residue identifier.
> The surface generated using **-i**
> is not always the same as the
> surface generated by running the entire molecule and afterwards
> selecting the desired atoms. The first surface will not include
> reentrant surface lying between an atom in *sitefile* and atoms not
> in the file.

> **-n**
>   
>  Include the unit normals to the surface with each surface point record.

> **-w** *radius*
>   
>  Change the "water" probe *radius* from the default of **1.4**
> angstroms. *Radius* must be between **1.0** and **201.0**.

> **-v**
>   
>  Produce more verbose output. **Dms**
> will announce each computation phase
> as it is entered as well as a count of the atom types in the molecule
> and the number of computation requests handled by each host that
> participated in the calculation.

> **-o** *outfile*
>   
>  The output surface is written to *outfile*.
> This flag is not optional.

The output consists of a series of atom and surface point records, with the
same format for the first six fields. Each atom is followed by the surface
points (if any) which belong to it. These first six fields are in the
following format: residue name, sequence number, atom name, x coordinate,
y coordinate, z coordinate. For an atom record, the seventh field is "A."
For a surface point record, the seventh field begins with an "S,"
followed by a "C," "R," or "S" according to whether the point is part of
contact, reentrant, or saddle surface (saddle is a type of reentrant
surface where the probe is in contact with exactly two atoms). This
is followed a digit used for depicting different density levels. The
eighth field is the molecular surface area associated with the point in
square angstroms. If **-n** is specified, the
next three fields are
the unit normal vector pointing outward from the surface.
Messages and errors are written to the standard error output
unless a separate log file has been
specified with **-g**.

**Dms** reads the elements and radii from a file named **radii**.
If there is a file in the current
directory called **radii**, then **dms** will use that file instead.
To add uncommon elements or use different radii, one should copy the
default radii file from the distribution and modify it.
The file format is documented in the file itself.

#### SEE ALSO

Richards, F.M.,
"Areas, volumes, packing and protein structure,"
*Ann. Rev. Biophys. Bioeng.* **6**, 151-176 (1977).

#### AUTHOR

Conrad Huang
  
University of California, San Francisco

#### DIAGNOSTICS

Many and varied. Be sure to examine the output messages (standard error
or *logfile*, if specified) before leaving a background job running.
