# Supplementary material for: DrugOn: a fully integrated pharmacophore modeling and structure optimization toolkit
Source: PeerJ. 2015 Jan 13;3:e725. doi: 10.7717/peerj.725 (PMC4304849; doi:10.7717/peerj.725)
Supplement: Supplemental Information 1 [file peerj-03-725-s001.zip › DrugOn_setup/Supplementary_Files/opal-py-2.4.1/docs/index.html]

Python Opal Web Service Client


# Python Opal Web Service Client: Version 2.4

---

## 1. Overview

This distribution contains the WSDL for the Opal Web service toolkit,
and a generic Python client that can call any Opal2 service that is already
deployed. If you would like to install your own Opal services, you should
follow the documentation for the server-side installation of Opal here.

## 2. Prerequisites

The Python client should work on both Unix and Windows platforms as long
as you have the following packages installed.

1. Python 2.3 or higher: Download and install Python from http://www.python.org/, if you don't
   have a Python installation on your local machine. Add the Python bin
   directory to your environment variable PATH.

## 3. Installation

1. Download the Python source distribution for installation from here,
   if you don't have the appropriate version already.

   Expand the tarball that you have downloaded using the GNU tar utility,
   as follows:

   ```
       tar zxvf opal-py-$VERSION.tar.gz
   ```

   This should create a new directory called opal-py-$VERSION/ where all
   the sources are expanded. Henceforth, we will call this directory
   *OPAL\_PY\_HOME*.
2. For your convenience, we are including ZSI version 2.1\_a2 as part of
   this distribution. ZSI is a SOAP and Web services toolkit for Python.
   The ZSI version included with this distribution can also be obtained
   from the SourceForge SVN using *svn co --revision 1490
   https://pywebsvcs.svn.sourceforge.net/svnroot/pywebsvcs/trunk/zsi*.
   To use the Opal clients, you only need the *egg* version of the ZSI
   installation from the *prereqs* directory. However, if you need a
   full installation of ZSI (which is useful for features such as code
   regeneration from WSDL), you may install it from the tarball provided in
   the same directory.

   To access the client-side functionality of ZSI, you may simply add
   the ZSI-2.1\_a2-py2.6.egg file to your PYTHONPATH. If you are using a
   bash shell on Unix, you can execute:

   ```
       export PYTHONPATH=$OPAL_PY_HOME/prereqs/ZSI-2.1_a2-py2.6.egg:$PYTHONPATH
   ```

   If you are using a tcsh shell, you can execute:

   ```
       setenv PYTHONPATH $OPAL_PY_HOME/prereqs/ZSI-2.1_a2-py2.6.egg:${PYTHONPATH}
   ```

   **OPTIONAL**: If you wish to install the full version of ZSI, you would
   need to use a sudo or root shell on Unix. Expand the
   prereqs/zsi-2.1\_a2.tar.gz tarball, change to the directory that is
   extracted from it, and execute the following command:

   ```
      python setup.py install
   ```

   Note the location where the ZSI package is installed. In particular,
   note the location of the *wsdl2py* script that we may need to use
   in the future for code generation. Typically, the packages are installed
   inside the Python installation. On Windows, wsdl2py typically resides
   inside $PYTHON\_HOME/scripts, where PYTHON\_HOME represents the location
   of the Python installation. On Unix, it is usually inside
   $PYTHON\_HOME/bin.

   *Opal will not work with any other version of ZSI. Please ensure
   that you are using the versions shipped with this installation of Opal.*
3. **OPTIONAL**: If you wish to regenerate the client-side stubs, you can
   do so by using the Opal WSDL. However, you must have a full ZSI
   installation done manually as described above. Most users can use the
   pre-shipped stubs, and skip this step.

   To generate the stubs from the WSDL file, run the following command inside
   the $OPAL\_PY\_HOME directory:

   $WSDL2PY\_HOME/wsdl2py wsdl/opal.wsdl

   This generates two relevant files - AppService\_client.py and
   AppService\_types.py. The former contains the stubs required to
   communicate with the remote Web service, while the latter contains the
   types defined in the WSDL for the service.

## 4. Running the Generic Client

A generic Opal client is implemented by
$OPAL\_PY\_HOME/GenericServiceClient.py. It allows the user to launch Opal
jobs, query status and retrieve job outputs.

To get the complete usage of the generic Opal client, you can execute:

```
      python GenericServiceClient.py
```

The Opal python client supports the following arguments:

|  |  |
| --- | --- |
| -l <url> | service url |
| -a <args> | command line arguments |
| -b/-f <attch1,attch2,..> | local input files as binary attachments |
| -j <job\_id> | job ID |
| -n <num\_procs> | number of processors (parallel jobs only) |
| -r <operation> | remote operation to invoke: [getAppMetadata\|launchJob\|queryStatus\|getOutputs] |
| -u <url1,url2,..> | input file urls |

For example, to launch the remote PDB2PQR Opal Serivce on
http://ws.nbcr.net, you may use the
following command (assuming you have the input file
1hpx.pdb in your directory):

```
   python GenericServiceClient.py -l http://ws.nbcr.net/opal2/services/Pdb2pqrOpalService \
   -r launchJob -a "--ff=amber 1hpx.pdb output.pqr" -f 1hpx.pdb
```

The above command will
return a jobID that can be later used to query status and
retrieve locations of the output files. Alternatively, the service
can also be invoked in a *blocking* fashion with
-r launchJobBlocking, where the client
blocks until the remote execution is complete, and the outputs are
returned as a response to the same invocation.

## 5. Developers Guide

To use Opal with the python libraries you have to make sure that the ZSI
is installed properly (as indicated above) and the stubs and the client
(AppService\_client.py AppService\_types.py and OpalClient.py) are in your Python path. In this
section we provide some code snippets showing the main steps for invoking an
Opal service. The *url* should point to the
Opal application you want to invoke. argList is the command line as you would like to to invoke
your application and inputFile is a list of string containing the relative path to the local file
you want to upload. If you get an error regarding
importing "AppService\_client" then it is probably because the Python interpreter
can not find the stubs and the client (AppService\_client.py and AppService\_types.py).

```
import OpalClient

# Service URL
apbsURL = "http://kryptonite.nbcr.net/opal2/services/apbs_1.3"
client = OpalClient.OpalService(apbsURL)

# command-line arguments
argList = "apbs.in"
inputFile = ["etc/apbs.in", "etc/ion.xml"]

print "Launching remote Apbs job"
jobStatus = client.launchJobNB(argList, inputFile)
```

Once you get the pointer to the jobStatus you should pool the server
to check the exectution of your job with the following code.

```
while jobStatus.isRunning() :
    time.sleep(3)
    print "Polling job status"
    jobStatus.updateStatus()

if jobStatus.isSuccessful():
    print "Job execution finished sucessfully."
else:
    print "Job execution failed."
    print "Check the stdout.txt and the stderr.txt for errors...."
```

Finally you can read the pydoc to see a full description of the APIs.

### 5.2 Old Opal Python Client

If you still want to use the old Opal Python Client, which access directly
python classes produced by the stubs you can read this section to see some
example of coding.

First it is necessary to retrieve a reference to an
AppServicePort type, as shown below. The *url* should point to the
Opal application you want to invoke and you shold make sure you have the
stubs in your Python path (AppService\_client.py and AppService\_types.py).

```
import AppService_client
from AppService_types import ns0

appLocator = AppService_client.AppServiceLocator()
appServicePort = appLocator.getAppServicePort(url)
```

The second step to launch a job via Opal is to create the request message
as follows.

```
#creating the input request
req = AppService_client.launchJobRequest()
req._argList = "put the command line here"
req._numProcs = numberOfProcessors

#loading two files
# first file
inputFile1 = ns0.InputFileType_Def(’inputFile’)
inputFile1._name = "fileName1"
inputFile1._attachment = open("/some/path/fileName1", "r")
#second file
inputFile2 = ns0.InputFileType_Def(’inputFile’)
inputFile2._name = "fileName2"
inputFile1._attachment = open("/some/path/fileName2", "r")
inputFiles = [inputFile1, inputFile2]
req._inputFile = inputFile
```

Finally the client can invoke the remote application and wait for it's
termination. In this example, we present a polling approach to determine
when the execution of the application is finished. Alternatively, t is also
possible to use the *req.launchJobBlocking(req)* which blocks until
the end of the application.

```
#launch a remote job
resp = appServicePort.launchJob(req)
jobID = resp._jobID
code = resp._status._code

#wait till error (code 4) or completion (code 8)
while(code!=4 and code!=8):
    #sleep or wait
    code = appServicePort.queryStatus(AppService_client.queryStatusRequest(jobID))._code

#check job status for successful execution
if(status==8):
    #Job completed successfully
        resp = appServicePort.getOutputs(AppService_client.getOutputsRequest(jobID))
	#resp._stdOut  contains URL to stdout
	#resp._stdErr  contains URL to stderr 
	#resp._outputFile   contains a list of URL with all the other output
else:
    #Job died or something else went wrong
```

### 5.3 Security

The new OpalClient does not support yet security. So if you want to
use GSI you must use the old interface. The following
snippet of code shows how to access a secure Opal
service from Python.

```
# set the locations for the X509 certificate and key
cert = "location of X509 certificate"
key = "location of unencrypted private key"
...
    # example of ssl invocation
    appServicePort = appLocator.getAppServicePortType(
        baseURL + "opal/services/Pdb2pqrServicePort",
        ssl=1,
        transdict=dict(cert_file=cert, key_file=key),
        transport=httplib.HTTPSConnection)
...
```

Note that if you are using proxy certificates, they have to be RFC 3820
compliant. This can be done by creating a Grid proxy using
grid-proxy-init -rfc (only works for Globus Toolkit 4.0.x). This
has only been tested with OpenSSL version 0.9.7g, and may not work with
older versions.

## 6. Related Links

- More examples of client usage can be found at: NBCR Opal Client Wiki

## 7. Feedback

Please use the Opal Toolkit Tracker on
Sourceforge to report bugs and feature requests.

---
