# Supplementary material for: DrugOn: a fully integrated pharmacophore modeling and structure optimization toolkit
Source: PeerJ. 2015 Jan 13;3:e725. doi: 10.7717/peerj.725 (PMC4304849; doi:10.7717/peerj.725)
Supplement: Supplemental Information 1 [file peerj-03-725-s001.zip › DrugOn_setup/Supplementary_Files/opal-py-2.4.1/docs/OpalClient.html]

Python: module OpalClient

|  |  |
| --- | --- |
| **OpalClient** | index ../OpalClient.py |

**Modules**

|  |  |  |  |
| --- | --- | --- | --- |
| getopt  httplib | os  sys | tarfile  time | urllib2 |

**Classes**

JobStatus

OpalService

|  |  |  |
| --- | --- | --- |
| class **JobStatus** | | |
|  | This class represents a Opal job status and can be used after launching a job to   monitor its execution. A Job Status is returned by a launchJob call, or it can be   constructed from a jobID and its corresponding Opal Service | |
|  |  | Methods defined here:  **\_\_init\_\_**(self, opalService, jobID)  A JobStatus can be constructed passing a OpalService object and string   containing a valid jobID  **destroyJob**(self)  request the server to kill this job  **downloadOutput**(self, baseDir)  download all output files from the job and it places them in the local baseDir   (baseDir must exists). This function currently works only with newer opal services,     @return: true if the operation was successful false otherwise  **getBaseURL**(self)  it returns the URL that contains all the job outputs  **getError**(self)  It returns the error message of the job  **getJobId**(self)  it returns the jobid of this job  **getOutputFiles**(self)  it returns a list of strings containing the URLs of the output files   produced by the job  **getStatus**(self)  it returns the numeric representation of the status of the job  **getURLstderr**(self)  it returns the URL of the stderr  **getURLstdout**(self)  it returns the URL of the stdout  **isRunning**(self)  this function returns true is the job is still running false if it finished  **isSuccessful**(self)  If the job sucesfully finished its execution, this function returns true  **updateStatus**(self)  this function retrives a updated version of the jobStatus   from the Opal server |

|  |  |  |
| --- | --- | --- |
| class **OpalService** | | |
|  | This class wrap a single Opal service. You should have one of this class for each   Opal Service you want to use. | |
|  |  | Methods defined here:  **\_\_init\_\_**(self, url)  A OpalService can be contructed passing a string containing a valid Opal URL  **getServiceMetadata**(self)  return the XML appConfig used by this application  **getURL**(self)  @return: a string containing the end point URL used by this services  **isOpal2**(self)  it returns true if this service points to a opal2 server  false in the other cases  **launchJobBlocking**(self, commandline, inFilesPath, numProcs=None, email=None, passwd=None)  launchJobBlocking(commandline, inFilesPath[, numProcs, email, passwd]) -> JobStatus     This method functions like the launchJobNB, but instead of returning it waits  until the invoked application is finished or failed.  **launchJobNB**(self, commandline, inFilesPath, numProcs=None, email=None, passwd=None)  launchJobNB(commandline, inFilesPath[, numProcs, email, passwd]) -> JobStatus     It invokes the execution of the remote application and return.      commandline: is a string containing the command line that should be executed  inFilesPath: is a list of strings containing relative or absolute path to the       files need to be uploaded for the execution of the application  numProcs: is the number of processors that should be used to run the parallel       application (this option is valid only for parallel applicaiton)  email: is a string containing the email used to send notification when the       application execution will finish  passwd: is a string containing the passwd used to authenticate with the server     @returns: a JobStatus Oject which can be used to monitor its execution |
