# Supplementary material for: DrugOn: a fully integrated pharmacophore modeling and structure optimization toolkit
Source: PeerJ. 2015 Jan 13;3:e725. doi: 10.7717/peerj.725 (PMC4304849; doi:10.7717/peerj.725)
Supplement: Supplemental Information 1 [file peerj-03-725-s001.zip › DrugOn_setup/Supplementary_Files/sphgen_cpp/README.pdf]

SPHGEN_CPP

Andrew Magis
University of Florida

Sphgen_cpp is a modified form of the DOCK suite program Sphgen. It has been translated from the original Fortran into C++, and the following improvements have been made:

- Sphgen frequently crashed on large inputs. This was caused by either the hard-coded upper limit of surface points being exceeded, or irregularities in the column spacing of molecular surface file atom numbers, which were used as indices into arrays. Sphgen_cpp does not have these problems, as most of the arrays have been eliminated, and all memory is dynamically allocated.
- Some calculations have been optimized to improve performance, and most of the disk accesses have been eliminated. As a result, sphgen_cpp runs approximately 2X to 2.5X as fast as Sphgen, depending on the input size.
- Use of the INSPH file is now optional, as command-line arguments are now accepted. Details are below.

To make Sphgen_cpp, type:

%>make

To clean up executable and object files, type

%>make clean

To run Sphgen_cpp, the following command line inputs are now accepted:

-i <inputfilename>
    The name of the molecular surface file to be read
-o <outputfilename>
    The name of the output sphere file to be written
-s [L|R] *(optional)*
    If R is specified, spheres are generated outside the receptor. If L is specified, the normal vectors of the MS file are inverted, and spheres are generated inside the receptor. Default is R.

-d [X|#] **Not currently implemented.**
Specifies subset of surface points to be used. Defaults to X.
-l [float] *(optional)*
Prevents generation of large spheres with close surface contacts. Defaults to 0.0
-m [float] *(optional)*
Minimum sphere radius in Angstroms. Defaults to 1.4
-x [float] *(optional)*
Maximum sphere radius in Angstroms. Defaults to 4.0

If no command line inputs are specified, the program looks for a file called INSPH in the working directory. INSPH should be formatted the same as for the original sphgen. Please note that while only the input and output file names are required if command line inputs are used, the INSPH file must contain everything.

Slight output differences exist between sphgen_cpp and sphgen. Occasionally a cluster will be output with one more or less sphere, and rarely a few spheres will be different. I attribute this to slight calculation and rounding differences between Fortran and C++, resulting in some spheres being included in clusters or not depending on if the difference crosses a user-specified limit or not. This should have a negligible effect on docking calculations.

Sphgen_cpp has been compiled using the GNU g++ compiler and tested on Linux CentOS 4.1 and Solaris.

Note: I have not implemented the surface point subset command –d, because I don't know what it is used for. If you use it, want to see this functionality, and can explain to me what this is used for, then please send me an email.

This software should be considered in beta testing. It probably has bugs. If you find one, or if you have any questions, comments, or suggestions, feel free to contact the author at andrewmagis@gmail.com.
